# Drug screening using model systems: some basics

**DOI:** 10.1242/dmm.028159

**Published:** 2016-11-01

**Authors:** Ross Cagan

**Affiliations:** Icahn School of Medicine at Mount Sinai, Annenberg 25-40, Campus Box 1020, 1468 Madison Avenue, New York, NY 10029, USA

## Abstract

An increasing number of laboratories that focus on model systems are considering drug screening. Executing a drug screen is complicated enough. But the path for moving initial hits towards the clinic requires a different knowledge base and even a different mindset. In this Editorial I discuss the importance of doing some homework before you start screening. 'Lead hits', 'patentable chemical space' and 'druggability' are all concepts worth exploring when deciding which screening path to take. I discuss some of the lessons I learned that may be useful as you navigate the screening matrix.

## Abstract (precis)

**Summary:** The author provides some tips on what to consider before you begin drug screening.

## The path to drug discovery

Recently, an increasing proportion of scientists, taxpayers and funding agencies have emphasized the importance of moving basic science towards the clinic. Simple model systems provide a powerful tool for generating and exploring new concepts, and new therapeutics. But the world of therapeutics is different from, say, the world of fly genetics: the two worlds speak a different language and, more importantly, they make different assumptions. The path to drug development for academic scientists can be daunting, even *after* identifying a therapeutic candidate that you have decided is worth pursuing.


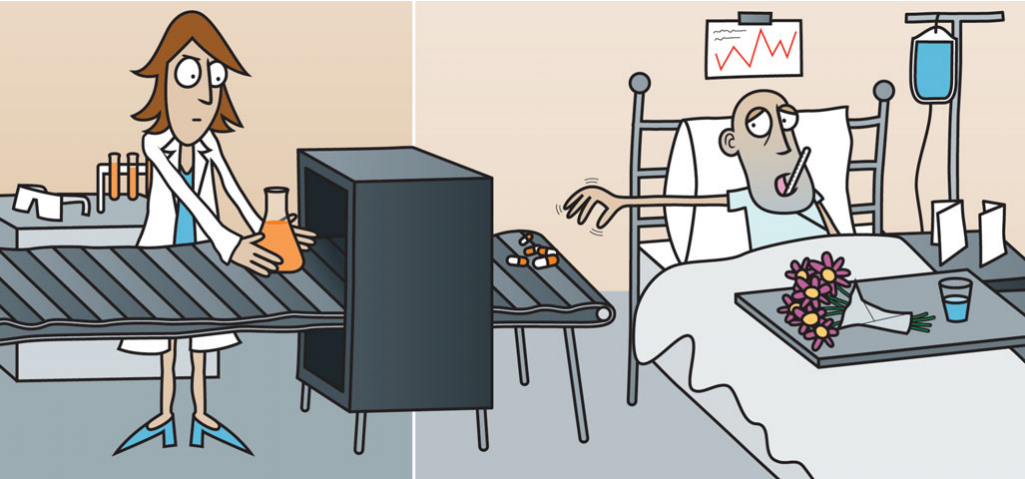


When my laboratory entered the world of drug discovery and drug development, I knew basically nothing about ‘patentable clinical space’, ADME studies (Box 1), or why screening natural compounds might not always be a great idea. I assumed that if we found drugs that ‘cured’ our fly disease models and then accomplished the same in the mouse, the drug would magically enter clinical trials. Somehow. Because it made sense.

**Box 1. ADME properties**

Once you have identified a promising lead, you will need to begin thinking about identifying its ADME (absorption, distribution metabolism and excretion; see below) properties. There are several contract research organizations (CROs) that will provide a preliminary assessment of the ADME properties of your compound for a reasonable amount of money. Larger scale ‘investigational new drug (IND)-enabling’ studies will be considerably more expensive. Having ADME data will change the level of conversation you will have with potential partners and investors.

The acronym stands for:

**A**bsorption: how readily the compound enters the bloodstream. Poor absorption means poor ‘bioavailability’.

**D**istribution: how readily the drug makes it through the body and to the target tissue. For example, if your compound strongly binds to blood albumen it may not be available to enter cells.

**M**etabolism: how quickly your compound is cleared from the bloodstream. Up to a point, longer is better, preferably hours. A compound that is clear in minutes by P450 enzymes in the liver will need to be altered.

**E**xcretion: this parameter measures how completely and efficiently the compound or its metabolites is cleared from the body.

Having a lead compound that passes all of these ADME properties is unusual. This is where having backup analogs is useful, and another reason to have chemists as friends.

My goal in this piece is not to review how to screen drugs using model systems – there are plenty of excellent reviews on this subject already (Phillips and Westerfield, 2014; Gopinathan et al., 2015; Asnani and Peterson, 2014; Veinotte et al., 2014; Lasserre et al., 2015; Simon and Bedalov, 2004; Kaletta and Hengartner, 2006; Yadav et al., 2016). My goal is to share some of the knowledge my group has gained over the years on how to *think about* drug screening. If you are asking the same question I asked a decade ago – “Why didn't anyone explain this to me *before* we started?” – this Editorial is for you.

## Getting started

The first question you need to ask yourself is: why do I want to do drug screening? If the answer is “to build tools” for exploring basic biology, then specificity trumps druggability. If it is “to satisfy funding agencies”, well, good luck with that. Here, I focus on researchers who have the goal of developing treatments for patients. Let's start with some basic terms:

**Drug versus compound:** pharma types are sticklers on this point. *Drugs* are approved for patient use; in the United States that means approved by the Food and Drug Administration (FDA). If you are using a chemical library your *hits* are *compounds*; your next step is to mature that initial compound into a *lead compound* or *lead hit*.

**Therapeutic index (TI):** this is the ratio of *efficacy* (how effective is the drug?) and toxicity (how toxic?) at optimal doses. This often leads to a discussion of the *therapeutic window* of the compound, i.e. the dose range at which a compound helps the patient without significantly harming them.

**Druggable space:** does the structure of your compound look like other drugs? Some structures are known to be toxic, and most chemical structures are too large, too small, or just do not look like they would have biological activity or that a chemist could easily make more of it (see ‘natural compounds’ below). Instead, you want to work in *druggable space*; that is, the compounds in your library roughly look like drugs that work in people. Incidentally, you will hear mention of *Lipinski's rule of five*; I recommend you look this one up (Box 2), then casually drop the phrase next time you talk to a chemist.

**Box 2. Lipinski's rule of five**

Christopher Lipinski, a Pfizer chemist, developed this rule in 1997 to identify characteristics commonly found in successful drugs. The rule states that a successful lead should have at least three of the following four (this is *rule* of five, not five rules) properties:

Five or fewer hydrogen bond donors
  Ten (2×5) or fewer hydrogen bond acceptors
  The compound should be small, typically less than 500 Da
  The compound should be mildly lipophilic (the ‘five’ comes from the requirement that the octanol-water partition coefficient have a log value less than 5.
Pharmaceutical companies are increasingly willing to consider compounds that violate this rule, but this is information that you should be ready to supply if conversations advance.

## Curing the disease without killing the patient

If you have identified a target and you want to identify a drug, the first objective is to check whether a company is interested in developing a lead. Drug companies are amazing at developing targeted therapies: some will screen millions of compounds, and they have teams of computational chemists to help. Another important check: is your target likely to be the best therapeutic candidate for the disease? The driver of a disease is not necessarily the best therapeutic target, and the potential for toxicity is a key consideration. Most drug delivery systems – commonly an injection or pill – drug the whole body. Will the patient be OK if your target is partially suppressed throughout the body? Fully suppressed? By some estimates, most drugs fail owing to toxicity, not poor efficacy. Companies are increasingly pushing for knockdown of candidate targets in adult mice so that any toxic effects at the organismal level can be fully recognized.

In general, be prepared to honestly and frankly assess the strengths and weaknesses of each of the platforms that you used to validate your lead. For example, success in a mouse model is a good start, but you should be prepared to discuss the statistics of how predictive this is for clinical success. Many companies and investors like to see leads that show good efficacy in a variety of models. Be sure to benchmark your lead against the current standard-of-care therapeutic.

## Imagine the clinical trials

Planning the path to clinical trials is one of the hardest lessons to grasp when embarking on a therapeutics screen; it certainly was for me. If you have not fully explored the path by which your best lead hits will enter clinical trials, please stop screening. Clinical trials are expensive and time-consuming – they can require tens or even hundreds of millions of dollars and many years to implement. Some questions to consider:

**Who will run the clinical trials?** For major diseases, pharmaceutical companies or large biotechnology companies will most often finance and run later-stage trials. Getting their attention can be difficult, although liaisons between pharma and research institutes are becoming more common, especially for drug targets. Remember, most drug companies do not have sufficient resources to develop all of their own promising leads, for which they fully own the intellectual property (IP). The IP of your lead compound is typically owned by your institution, so you are already facing an uphill battle to attract interest.

One great piece of advice that I was given is to engage scientists at drug companies early on, even before you begin screening. Their scientists are smart, savvy about drug screening (especially the chemists) and often happy to give advice. Developing a relationship can be helpful throughout the screening process, and you are less of an unknown when you later have leads to discuss.

**Is there sufficient need for a new therapeutic?** Most diseases would benefit from improvements to available therapeutics, but you should be aware that approved therapeutics raise the bar on how great your therapeutic needs to be. Some diseases have several approved therapies; even if these therapies are not perfect, their existence will make investors less likely to invest in a new candidate.

**The special case of orphan diseases:** a great role for basic researchers is to develop drugs for orphan/rare diseases, defined in the United States as affecting fewer than 200,000 people. Although drug companies are taking on more orphan diseases, there are thousands of diseases in which people need help but the disease is too rare to attract attention. Fortunately, criteria for clinical trials are designed to attract interest into this space, so the trials are quicker and cheaper. Importantly, make sure that enough patients can be located to run a trial. Many trials fail due to lack of patient recruitment, and any investor considering your lead will want to hear that you have thought about this.

## Choosing a library

One of the most important and difficult decisions you will make is choosing a library to screen. When my laboratory started down this road, some kind scientists and chemists from pharma patiently explained some basic principles, for which I am eternally grateful. Please note there are varying opinions on the best libraries to use, and the following points are meant to stimulate an awareness of some the issues to consider.

**FDA-approved drugs:** one popular approach is to screen libraries of FDA compounds. The Center for Personalized Cancer Therapeutics at Mount Sinai uses a library of 1200 FDA-approved compounds to identify patient-specific cancer therapies. There are many technical issues to consider, beyond cost. For example, DMSO – the commonly used drug solvent – is toxic to *Drosophila* at fairly low concentrations, so most libraries are too dilute for our needs. Some tinkering before you purchase is important.

The advantage of identifying an FDA-approved drug is speed to patient. Issues relating to druggability, dosing and other parameters have already been worked out. Furthermore, if you are associated with a hospital, your colleagues may be able to establish a small early-stage trial fairly quickly. In the case of very rare diseases, for which finding enough patients to achieve statistical significance is not possible, FDA-approved drugs may provide the most efficient path forward.

However, FDA library screens also highlight some of the frustrations that basic researchers can have with the screening landscape. Most FDA-approved drugs will be off-patent by the time they would be approved in new trials, so the pharma industry is less excited to run an expensive clinical trial only to be faced with competition from generics. Remember that the goal of pharmaceutical companies is not to put themselves out of business. My personal experience is that they are usually generous in supplying drugs and know-how. The responsibility is ours to provide them with a way forward, by choosing our screening library wisely, and by being prepared to discuss the chemistry of our leads in an informed and sophisticated manner.

Which brings us to **compound libraries:** when selecting a library, remember that free is not always best. For example, some academic libraries emphasize ‘broad chemical space’, that is, lots of chemical variety. Avoid these. You want a library of chemicals that ‘sit in druggable space’ (for example, see Box 2 on Lipinski's rule of five). Importantly, you also want a chemical library in which each compound is backed by additional related analogs (usually available for a small fee), allowing you to ‘explore the local chemical space’ surrounding your initial hit. This is key both to generate better hits in your model system and also to give you more compounds to work with, for example, as you explore human cell lines. Also, investors like to know that you have backup compounds should your lead have fundamental flaws such as poor solubility.

**Drug cocktails:** cocktails provide a great opportunity for ‘polypharmacology’, i.e. to hit multiple therapeutic targets. My laboratory is heavily invested in the idea that diseases are usually complex, and can often benefit from targeting multiple parts of a disease network through polypharmacology. However, if you are looking to create drug cocktails, make sure you explore the IP issues. Getting two companies to collaborate on a clinical trial is becoming somewhat more common, but remains the exception. There are other issues to consider, such as unexpected drug interactions and the requirement for higher manufacturing tolerances. If you are looking to pair your novel drug with an approved drug, the same IP challenges emerge: will the company support the trial? Better to find out early. Also, notice how IP issues keep emerging. If you are screening for therapeutics and you want to help patients, become an expert in IP.

A side note here about targets: efficacy is the most important consideration, but knowing the direct targets and mechanism of action of your lead will help gain the attention of chemists. My experience is that many industry partners are only interested in drugs that hit a small number of defined targets; they most often prefer a ‘clean’ lead that hits a single target. I would like that to change – many of the most successful drugs in the clinics are not clean – but pharma continues to evolve slowly on this point.

**Natural compounds – a good idea?** When I first explored drug screening, many of my academic colleagues strongly promoted screening natural compounds. My drug company peers are not so enthusiastic. Most natural compounds are complex molecules that are difficult to synthesize to patient grade in a laboratory. As one colleague explained: “We spent 20 years learning how to synthesize taxol. We are not doing that again”. While that is a bit extreme, remember that the more difficult you make the chemistry, the lower the likelihood is that your lead hits will move forward. Even in fields with large markets, most clinical trials fail, so the months or years of effort to synthesize a complex molecule can be difficult to justify.

## **Your** **first hit is a great start, but just a start**

This section could just as easily be labeled ‘make friends with chemists’. In fact, while I'm thinking about it, a shout out to my chemist friends. (Always be nice to chemists!) In return, they will:

- tell you if your drug is in fact in druggable space
- move your drug into patentable chemical space
- make analogs to ‘play in the chemical space’
- properly speak to chemists when you engage pharma
- help you write a patent to protect your lead hit
- identify a corporate research organization for preliminary ADME studies, the first step towards investigational new drug (IND)-enabling studies.
- read your commentaries and make helpful suggestions (thanks Arvin).

I have been fortunate to work with smart chemists who are also interested in the biology, including Arvin Dar, Bob Devita, Avner Schlessinger and Kevan Shokat. If you can find chemists who will work with you to the level of understanding the biology, then set up regular meetings and listen to what they have to say.

## IP and patentable chemical space

I will not tackle the intricacies of IP here, but let's talk about some basic points. You have screened and you have a hit. You don't care at all about making money; indeed, the idea makes you uncomfortable and you worry that it will skew your science. You should file that patent anyway.

If you have not – or cannot – patent your lead compound, then patients will not benefit from your work. In fact, if you publish the compound without patenting it you are actively preventing anyone from benefiting therapeutically by blocking the ability of the chemical space to be protected. No intellectual property, no clinical trial. Unless you have the millions of dollars required to move the drug forward by yourself, get it patented.

## The take-home message

Drug discovery can be a rewarding outcome to a line of research. My best advice is to be intentional about each step. Research the whole road forward, and engage experts. Often, the rules of engagement are not intuitive. Chemists and people in pharma can speak a different language from your academic colleagues, in part because they understand the long and complex road required for success. But they are often willing to mentor because, like the rest of us, they want to help patients. By doing a bit of homework before you start screening, you can ensure that your ‘lead hit’ sits in ‘patentable chemical space’, creating a smoother path to clinical trials.
